# Caregivers' burden and its influencing factors among parents of children with asthma and comorbidities: a cross-sectional study

**DOI:** 10.3389/fpubh.2025.1698723

**Published:** 2025-11-07

**Authors:** Xin Yi, Kejimu Sunzi, Jie Liu, Fang Yang, Xia Wu, Dan Bi, Qin Huang, Lifei Dai

**Affiliations:** 1 School of Basic Medical Sciences & School of Nursing, Chengdu University, Chengdu, China; 2 Department of Pediatrics, Deyang People's Hospital, Deyang, China; 3 Department of Psychosomatic Medicine, Deyang People's Hospital, Deyang, China; 4 Department of Nursing, Deyang People's Hospital, Deyang, China; 5 Department of Emergency, Deyang People's Hospital, Deyang, China

**Keywords:** asthma, comorbidity, caregiver burden, parents, caregivers

## Abstract

**Objective:**

This study aims to investigate the current status of caregiver burden among parents of children with asthma comorbidities and to identify its associated factors.

**Methods:**

We conducted a cross-sectional study from May to July 2025, involving 325 parents of children with asthma and comorbidities from two tertiary hospitals in Sichuan Province, China. Sociodemographic and clinical characteristics were collected through structured questionnaires. The Zarit caregiver burden interview (ZBI) and perceived social support scale (PSSS) were used to assess caregiver burden and social support levels, respectively. Statistical analyses included Pearson correlation, *t*-tests, analysis of variance (ANOVA), and multiple linear regression.

**Results:**

The total caregiver burden score was 40.44 ± 6.86, with 61.8% of the parents experiencing a moderate level of burden. Perceived social support was negatively correlated with caregiver burden (*r* = −0.621, *P* < 0.01). Multiple linear regression analysis revealed the child's age, asthma course, asthma comorbidity course, number of comorbidities, payment methods of healthcare costs, employment status, asthma control status, and perceived social support as factors significantly associated with caregiver burden, collectively explaining 55.2% of the total variance. This indicates that nearly half of the variance in caregiver burden could not be explained.

**Conclusions:**

The caregiver burden among parents of children with asthma and comorbidities is substantial. Factors associated with higher burden levels include longer asthma course, greater number of comorbidities, longer comorbidity course, part-time employment status, and poor asthma control. Conversely, higher levels of perceived social support, older child age, and medical insurance and commercial insurance coverage were associated with lower burden levels. These findings provide an empirical basis for clinical institutions, communities, and relevant departments to inform the development of targeted interventions aimed at alleviating this caregiver burden. It should be noted that this study employed a cross-sectional design, and future longitudinal research is needed to verify causal relationships.

## 1 Introduction

Asthma is a heterogeneous disease characterized by chronic airway inflammation and heightened airway responsiveness, clinically manifested as recurrent episodes of wheezing, coughing, and shortness of breath, which often occur or worsen at night or in the early morning (1). As a chronic airway hyperresponsiveness condition that severely affects the physical and mental health of children worldwide, the prevalence of pediatric bronchial asthma has been increasing (2, 3), posing a substantial burden on global public health systems. According to the 2021 Global Initiative for Asthma (GINA) report, asthma affects over 300 million children globally (4), posing a critical and increasing public health burden that demands urgent attention worldwide (5–7).

In recent years, clinical practice and research have revealed that childhood asthma is often accompanied by comorbidities, a situation that further increases the complexity and challenges of disease management. In 2008, the World Health Organization (WHO) defined “comorbidity” as the coexistence of two or more chronic non-communicable diseases in the same patient (8). Common comorbidities of childhood asthma include allergic rhinitis (AR), chronic rhinosinusitis (CRS), and obstructive sleep apnea (OSA), among others (9). Studies indicate that 85%−95% of children with asthma have comorbid AR (10), 63% have comorbid OSA (11), and 49.2% experience anxiety-related issues (12). These comorbidities not only directly and negatively impact the condition of children with asthma, such as increasing the difficulty of asthma diagnosis and reducing treatment effectiveness (13, 14), but also lead to a higher frequency of asthma exacerbations, as well as increased emergency department visits and hospitalizations (13, 15), substantially consuming medical resources. Furthermore, the long-term management of comorbidities requires parents to invest more time in monitoring multiple disease symptoms and coordinating multidisciplinary treatments. Simultaneously, higher treatment costs and concerns about the child's prognosis impose significant physical, psychological, and economic pressures on parents (16). Given that caring for children with asthma and comorbidities is a long-term and continuous process, and that parents play a critical role in disease management, with the quality of care they provide determining the disease progression and recovery of the child, it is crucial to recognize and address the caregiver burden experienced by parents of children with asthma comorbidities.

Caregiver burden refers to the negative reactions experienced by caregivers in physical, psychological, and economic aspects due to the perceived stress of providing care to patients (17). Studies have shown that caring for children with chronic diseases presents greater challenges than caring for healthy children, and the high-quality care required by affected children is more likely to impose a heavy burden on caregivers (18). As the primary caregivers of children with asthma (19), parents bear multiple disease-related pressures, and the presence of asthma comorbidities further exacerbates this burden. On the physical level, caring for children with asthma comorbidities requires parents to expend considerable time and energy over the long term: asthma comorbidities are prone to triggering acute episodes or causing persistent symptoms in children, thereby increasing the need for emergency visits and hospitalizations (20). Parents must accompany their children throughout diagnosis and treatment, and this significant time investment often leads to physical fatigue and exacerbates exhaustion (21). On the psychological level, long-term care commonly leads to anxiety and depressive symptoms among parents (22). Studies report that 67.57% of parents of children with asthma experience varying degrees of depression (23). Additionally, concerns about the child's physical and mental impairment, uncertainty about disease prognosis, and pressures from social and work responsibilities further compound their psychological and emotional burden. On the economic level, children with asthma comorbidities require more complex health management and greater use of medical services. Long medication cycles and high treatment costs (16), coupled with the fact that comorbidities increase asthma-related treatment expenses (24), place a substantial financial strain on families.

Given that asthma comorbidities severely affect children's health and impose multiple burdens on their caregivers, addressing parental caregiver burden in this population is crucial. However, existing literature primarily focuses on asthma as a single disease (25–28), thereby overlooking the cumulative impact of multiple comorbidities. Specifically, there is a notable lack of studies investigating how the coexistence of several comorbidities amplifies caregiver burden, creating a significant gap in understanding the challenges faced by parents of this complex patient population. To address this gap, this cross-sectional study aims to investigate the current status of caregiver burden and its associated factors among parents of children with asthma and comorbidities, and employs the total number of comorbidities per child to operationalize this cumulative impact in our analysis. The findings will provide an empirical basis for developing targeted intervention strategies aimed at alleviating this caregiver burden and improving disease management outcomes.

## 2 Materials and methods

### 2.1 Survey participants

In this study, A convenience sampling method was employed to recruit parents of children with asthma and comorbidities who visited the pediatric outpatient departments of two Grade A tertiary hospitals in Sichuan Province from May 2025 to July 2025 as participants.

Inclusion criteria: parents of children diagnosed with asthma and at least one comorbidity (including allergic rhinitis, chronic sinusitis, obesity, gastroesophageal reflux disease, obstructive sleep apnea, anaphylaxis, anxiety and depression issues) based on the diagnostic criteria specified in Guidelines for the Diagnosis and Management of Bronchial Asthma in Children by the Respiratory Subspecialty Group of the Pediatrics Society, Chinese Medical Association (1); parents of children aged 0–18 years; parents with normal thinking and verbal communication abilities, as well as adequate reading and comprehension skills; parents who voluntarily agreed to participate in the study and signed the informed consent form.

Exclusion criteria: parents with mental illness or other severe chronic conditions that prevented them from completing the questionnaire independently; parents or their children who had experienced major life changes or stressful events (e.g., the death of a family member, a serious traffic accident, etc.) within the past 3 months.

Based on Kendall's rough estimation principle for sample size, the sample size of a study should be at least 5–10 times the number of variables (29). In this study, a total of 25 variables were included. Accounting for potential invalid questionnaires, the sample size was increased by 10%−20%, resulting in a calculated required sample size of 138–300 participants. To ensure more reliable statistical results, we attempted to collect as much data as possible. Ultimately, 340 questionnaires were distributed in this study, with 325 valid ones recovered, yielding an effective response rate of 95.59%.

This study has obtained approval from the Ethics Committee of Deyang People's Hospital (No. 2025-04-043-K01) and the Medical Ethics Committee of the Affiliated Hospital of Chengdu University (No. PJ2025-056-03). All participants were fully informed about the study, provided their informed consent, and voluntarily participated.

### 2.2 Survey instruments and measures

#### 2.2.1 General information questionnaire

The questionnaire consists of two sections: one for the children and one for their parents. The child's general information questionnaire includes items such as gender, age, duration of asthma, duration of asthma comorbidities, asthma control status, number of comorbidities, family history of asthma, medical expense payment method, and the child's annual medical expenses. The parents' general information questionnaire covers items like gender, age, educational level, marital status, number of children, employment status, and monthly family income.

#### 2.2.2 Zarit caregiver burden inventory (ZBI)

The Zarit Caregiver Burden Inventory (ZBI), developed by Zarit et al. (17), is used to assess the level of burden experienced by caregivers. The ZBI questionnaire used in this study was translated and revised by Chinese scholar Wang et al. (30). The Chinese version of the questionnaire consists of 22 items, divided into two dimensions: personal burden and responsibility burden. Specifically, it includes 12 items related to personal burden (Items 1, 4, 5, 8, 9, 14, 16–21) and 6 items addressing other forms of burden (Items 2, 3, 6, 11–13). Items 7, 10, and 15 are not categorized under any dimension, while Item 22 serves as an overall evaluation of the caregiver's perceived burden. The questionnaire uses a 5-point Likert scale, with responses ranging from 0 to 4, corresponding to “never”, “rarely”, “sometimes”, “often”, and “always” respectively. Total scores range from 0 to 88, with higher scores indicating greater caregiver burden. Scores of 0 to 19 indicate no burden, 20 to 39 indicate mild burden, 40 to 59 indicate moderate burden, and scores above 60 indicate severe burden. The Cronbach's alpha coefficient of the original scale is 0.870, and in the present study, the Cronbach's alpha coefficient for the scale was 0.832.

#### 2.2.3 Perceived social support scale (PSSS)

The perceived social support scale (PSSS), developed by Zimet et al. (31), is used to assess an individual's level of perceived social support. The PSSS questionnaire used in this study was translated and revised by Chinese scholar Jiang et al. (32). The Chinese version of the questionnaire consists of 12 items across three dimensions: family support, friend support, and other support. Specifically, there are 4 items for family support (Items 3, 4, 8, 11), 4 items for friend support (Items 6, 7, 9, 12), and 4 items for other support (Items 1, 2, 5, 10). The questionnaire uses a 7-point Likert scale, with responses ranging from 1 to 7, corresponding to “strongly disagree”, “disagree”, “slightly disagree”, “neutral”, “slightly agree”, “agree”, and “strongly agree” respectively. Total scores range from 12 to 84, with higher scores indicating a higher level of perceived social support. Scores of 12–36 indicate a low level of perceived social support, 37–60 indicate a moderate level, and 61–84 indicate a high level. The Cronbach's alpha coefficient of the original scale is 0.840, and in the present study, the Cronbach's alpha coefficient for the scale was 0.814.

#### 2.2.4 Data collection

This study employed a convenience sampling method, which is a core type of non-probability sampling. Convenience sampling is characterized by the accessibility and ease of participant selection, allowing researchers to prioritize individuals who are readily available, easily recruited, and meet the inclusion criteria in a specific research setting, without adhering to randomization principles to cover all individuals in the population (33). The reasons for selecting convenience sampling are as follows: first, as a cross-sectional study requiring sample collection within a limited timeframe, this method effectively reduces the difficulty of sample screening and shortens the data collection cycle. Second, for cross-sectional studies focusing on target populations in specific settings, this approach ensures that enrolled participants can provide the core information required for the research, balancing feasibility and data completeness. It should be noted that while this method offers advantages in efficiency and practicality, its reliance on convenience rather than randomization may limit sample representativeness, thereby restricting the generalizability and broader applicability of the findings. Participants were selected in strict accordance with the predefined inclusion and exclusion criteria. Questionnaires were collected through on-site surveys with immediate retrieval. To ensure consistency in the research process, the research team first provided uniform training to its members on interview techniques, key points for questionnaire completion, and relevant protocols. Before distributing the questionnaires, researchers thoroughly explained the purpose and significance of the study to the participants. Questionnaires were only distributed after participants had a clear understanding of the study and signed the informed consent form. During questionnaire completion, participants could ask researchers questions at any time; researchers provided on-site explanations but only for clarification purposes, avoiding suggestive or deliberate guidance and refraining from interfering with participants' responses. Once the questionnaires were completed, researchers collected them on the spot and reviewed the responses. If any errors or omissions were identified, participants were immediately asked to supplement or correct the information to minimize data loss.

### 2.3 Statistical analysis

Data from the questionnaires were entered using Microsoft Excel 2019 to establish and organize the database. Statistical analyses were performed with SPSS 25.0 software. During the data cleaning phase, following an overall assessment of data quality, missing values were addressed using listwise deletion. Only complete cases without any missing values were included in the analysis to ensure both the sample size and reliability of subsequent analyses. Qualitative data are presented as frequencies and percentages. For quantitative data, the descriptive approach was determined based on their distribution. The normality of continuous variables was assessed using the Shapiro-Wilk test. Quantitative data that were normally or approximately normally distributed are described as means and standard deviations, while those not meeting the normality assumption are reported as medians and interquartile ranges. Independent samples *t*-tests and one-way analysis of variance were used to examine differences in caregiver burden scores among parents of children with asthma comorbidities based on demographic characteristics of both the parents and the children. Pearson correlation analysis was conducted to explore the correlation between parental caregiver burden and perceived social support, as well as the relationships between numerical demographic variables and caregiver burden. Multiple linear regression analysis was employed to identify factors influencing the caregiver burden among parents of children with asthma comorbidities. Nominal variables were converted into dummy variables before being entered into the regression model. Multicollinearity among the independent variables was assessed using the variance inflation factor (VIF) and tolerance to ensure the stability and accuracy of the regression model. A VIF < 10 and a tolerance > 0.1 indicated the absence of severe multicollinearity, allowing for the regression analysis to proceed. If VIF ≥10 or tolerance ≤ 0.1, remedial measures such as removing redundant variables or merging variables were applied before further analysis. The independence of residuals was verified using the Durbin-Watson test. To quantify the magnitude of the associations, the effect size (standardized coefficient β) along with its 95% confidence interval (CI) are reported. A *P*-value < 0.05 was considered statistically significant.

## 3 Results

### 3.1 Demographic characteristics of children with asthma comorbidities

Among 325 children with asthma comorbidities, there were 207 boys (55.50%) and 118 girls (39.30%). The average age of children was (6.43 ± 2.49) years. Course of asthma < 1 year: 90 (27.70%), 1–2 years: 75 (23.10%), ≥3 years: 160 (49.20%). Course of comorbidities of asthma < 1 year: 65 (20.00%), 1–2 years: 100 (30.80%), ≥3 years: 160 (49.20%). In terms of the number of comorbidities, there were 237 children (72.90%) who had 1 comorbidity, 84 (25.80%) had 2 comorbidities, and 4 (1.20%) had ≥ 3 comorbidities. There were 181 (55.70%) with a family history of asthma and 144 (44.30%) without such a history. See Table 1 for details.

**Table 1:** Demographic characteristics of children with asthma comorbidities (*N* = 325).

**Item**	**Category**	***N***	**Percentage (%)**
Child's gender	Male	207	63.70
	Female	118	36.30
Child's age (year)	< 5	69	21.20
	5–9	216	66.50
	≥10	40	12.30
Asthma course (year)	< 1	90	27.70
	1–2	75	23.10
	≥3	160	49.20
Asthma comorbidity course (year)	< 1	65	20.00
	1–2	100	30.80
	≥3	160	49.20
Asthma control status	Poorly controlled	144	44.30
	Partly controlled	156	48.00
	Well controlled	25	7.70
Number of asthma co-morbidities	1 comorbidity	237	72.90
	2 comorbidities	84	25.80
	≥3 comorbidities	4	1.20
Family history of asthma	Yes	181	55.70
	No	144	44.30
Annual medical expenses of the child (CNY)	< 3,000	49	15.10
	3,000–4,999	198	60.90
	≥5,000	78	24.00
Payment methods for healthcare costs	Self-funded	283	87.10
	Medical insurance	32	9.80
	Medical insurance and commercial insurance	10	3.10

### 3.2 Demographic characteristics among parents of children with asthma comorbidities

Among the 325 parents of children with asthma comorbidities, 113 were male (34.80%) and 212 were female (65.20%), with a higher proportion of females. The average age of the parents was (34.17 ± 3.57) years. In terms of educational level, 176 (54.20%) had a college education, 80 (24.60%) had a high school or technical secondary school education, 45 (13.80%) had a Junior high school or below, and 24 (7.40%) had a master's degree or above. Among the employment statuses, 234 parents (72.00%) were employed full-time, 24 parents (7.40%) were employed part-time, and 67 (20.60%) were in other employment statuses. See Table 2 for details.

**Table 2:** Demographic characteristics of parents of children with asthma comorbidities (*N* = 325).

**Item**	**Category**	***N***	**Percentage (%)**
Parents' gender	Male	113	34.80
	Female	212	65.20
Parents' age(year)	< 30	57	17.50
	30–39	245	75.40
	≥40	23	7.10
Education level	Junior high school or below	45	13.80
	High school or technical secondary school	80	24.60
	College education	176	54.20
	Master's degree or above	24	7.40
Marital status	Married	320	98.50
	Other	5	1.50
Number of children	1 child	211	64.90
	2 children	114	35.10
Employment status	Employed Full-Time	234	72.00
	Employed Part-Time	24	7.40
	Other employment	67	20.60
Monthly family income (CNY)	5,001–10,000	146	44.90
	10,001–15,000	167	51.40
	>15,000	12	3.70

### 3.3 Caregiver burden and perceived social support scores among parents of children with asthma comorbidities

The caregiver burden score among parents of children with asthma comorbidities was 40.44 ± 6.86, with 61.8% (*n* = 201) experiencing a moderate level of burden. The perceived social support score was 46.23 ± 4.20, indicating a moderate level of support. The scores of each dimension of the caregiver burden scale and the perceived social support scale. See Table 3 for details.

**Table 3:** Caregiver burden and perceived social support scores in parents of children with asthma comorbidities.

**Dimension**	**Scoring range**	**Scores**	**Sorting**
ZBI total score	0–88	40.44 ± 6.86	
Personal burden	0–48	23.02 ± 4.03	1
Responsibility burden	0–24	11.22 ± 2.60	2
Other burdens	0–16	6.20 ± 1.32	3
PSSS total score	12–84	46.23 ± 4.20	
Family support	4–28	16.27 ± 1.90	1
Friends support	4–28	15.06 ± 1.68	2
Significant other support	4–28	14.91 ± 1.44	3

ZBI, Zarit caregiver burden inventory; PSSS, perceived social support scale.

### 3.4 Correlation analysis of caregiver burden Score and perceived social support Score

Pearson correlation analysis revealed significant negative correlations between caregiver burden (total and dimension scores) and perceived social support (total and dimension scores) (all *P* < 0.01). Specifically, a strong negative correlation was found between the total scores of caregiver burden and perceived social support (*r* = −0.621, *P* < 0.01). See Table 4 for details.

**Table 4:** Correlation analysis of caregiver burden and perceived social support scores for parents of children with asthma comorbidities.

**Variable**	**Dimension**	**ZBI total score**	**Personal burden**	**Responsibility burden**	**Other burdens**
PSSS	PSSS total score	**–**0.621^**^	**–**0.590^**^	**–**0.564^**^	**–**0.315^**^
	Friends support	**–**0.523^**^	**–**0.473^**^	**–**0.494^**^	**–**0.302^**^
	Friends support	**–**0.495^**^	**–**0.503^**^	**–**0.422^**^	**–**0.204^**^
	Significant other support	**–**0.543^**^	**–**0.509^**^	**–**0.500^**^	**–**0.281^**^

^**^Indicates correlation is significant at the 0.01 level (two-tailed). ZBI, Zarit caregiver burden inventory; PSSS, perceived social support scale.

### 3.5 Univariate analysis of the caregiver burden among parents of children with asthma comorbidities

Univariate analysis identified several factors significantly associated with caregiver burden, including the child's age, asthma course, asthma comorbidity course, asthma control status, number of comorbidities, family history of asthma, annual medical expenses, payment methods of healthcare costs, parent's gender, educational level, employment status, and monthly family income (all *P* < 0.05). See Table 5 for details.

**Table 5:** Univariate analysis of caregiver burden among parents of children with asthma comorbidities (*N* = 325).

**Variables**	**Category**	***N***	**ZBI scores**	***t*/*F*/*r***	***P***
Child's gender	Male	207	40.50 ± 6.80	0.189^a^	0.850
	Female	118	40.35 ± 6.99		
Child's age (year)	< 5	69	43.59 ± 5.17	**–**0.218^c^	0.000
	5–9	216	39.65 ± 7.12		
	≥10	40	39.30± 6.51		
Asthma course (year)	< 1	90	39.06 ± 6.30	21.065^b^	0.000
	12	75	37.25 ± 6.82		
	≥3	160	42.72 ± 6.40		
Asthma comorbidity course (year)	< 1	90	40.20 ± 5.47	7.478^b^	0.001
	1–2	75	38.34 ± 7.63		
	≥3	160	41.86 ± 6.54		
Asthma control status	Poorly controlled	144	42.42 ± 6.07	12.358^b^	0.000
	Partly controlled	156	38.71 ± 6.83		
	Well controlled	25	39.88 ± 8.46		
Number of asthma comorbidities	1 comorbidity	237	39.18 ± 6.74	0.297^c^	0.000
	2 comorbidities	84	43.42 ± 5.64		
	≥3 comorbidities	4	53.00 ± 6.98		
Family history of asthma	Yes	181	41.50 ± 5.53	3.037^a^	0.003
	No	144	39.11 ± 8.06		
Annual medical expenses of the child (CNY)	< 3,000	49	40.71 ± 6.90	6.328^b^	0.002
	3,000–4,999	198	39.57 ± 7.13		
	≥5,000	78	42.49 ± 5.67		
Payment Methods for Healthcare Costs	Self-funded	283	41.01 ± 6.42	9.257^b^	0.001
	Medical insurance	32	38.09 ± 8.48		
	Medical insurance and commercial insurance	10	32.00 ± 6.98		
Parents'gender	Male	113	38.28 ± 7.50	**–**4.016^a^	0.000
	Female	212	41.59 ± 6.21		
Parents' age (year)	< 30	57	42.07 ± 5.26	**–**0.073^c^	0.190
	30~39	245	39.89 ± 7.09		
	≥40	23	42.30 ± 7.20		
Education level	Junior high school or below	45	43.62 ± 5.48	6.615^b^	0.000
	High school or technical secondary school	80	41.21 ± 6.43		
	College education	176	39.77 ± 7.01		
	Master's degree or above	24	36.88 ± 7.20		
Marital status	Married	320	45.40 ± 3.13	1.632^a^	0.104
	Other	5	40.37 ± 6.88		
Number of children	1 child	211	40.79 ± 6.44	**–**0.058^c^	0.298
	2 children	114	39.81 ± 7.57		
Employment Status	Employed full-time	234	39.74 ± 6.87	4.742^b^	0.009
	Employed part-time	24	43.08 ± 6.52		
	Other employment	67	41.96 ± 6.55		
Monthly family income (CNY)	5,001–10,000	146	41.81 ± 6.14	5.532^b^	0.009
	10,001–15,000	167	39.43 ± 7.17		
	>15,000	12	38.00 ± 8.26		

ZBI, Zarit caregiver burden inventory; ^a^*t*-value; ^b^*F*-value; ^c^*r*-value.

### 3.6 Multiple linear regression analysis of caregiver burden among parents of children with asthma comorbidities

Multiple linear regression analysis was performed with the total caregiver burden score as the dependent variable. The independent variables were those identified through univariate analysis and Pearson correlation analysis. The analysis revealed that perceived social support, the child's age, asthma course, number of comorbidities, payment methods of healthcare costs, asthma comorbidity course, employment status, and asthma control status were significantly associated with caregiver burden. These factors collectively explained 55.2% of the variance in caregiver burden (*R*^2^ = 0.552). The assignments of the independent variables are shown in Table 6, and the results of the multiple linear regression analysis are shown in Table 7.

**Table 6:** Assignment of independent variables.

**Independent variable**	**Assignment method**
Child's age	Continuous variables, direct inclusion
Asthma course (year)	< 1 (Reference group)
	1–2 = 1, ≥3 = 0
	≥3 = 1,1–2 = 0
Asthma comorbidity course (year)	< 1 (Reference group)
	1–2 = 1, ≥ 3 = 0
	≥3 = 1,1–2 = 0
Asthma control status	Uncontrolled (Reference group)
	Poorly controlled = 1, Partly controlled = 0, Well controlled = 0, Completely controlled = 0
	Poorly controlled = 0, Partly controlled = 1, Well controlled = 0, Completely controlled = 0
	Poorly controlled = 0, Partly controlled = 0, Well controlled = 1, Completely controlled = 0
	Poorly controlled = 0, Partly controlled = 0, Well controlled = 0, Completely controlled = 1
Family history of asthma	Yes = 1; No = 0
Number of asthma co-morbidities types	1 comorbidity (Reference group)
	2 comorbidities = 1, ≥3 comorbidities = 0
	≥3 comorbidities = 1, 2 comorbidities = 0
Parents'gender	Male = 0; Female = 1
Education level	Junior high school or below (Reference group)
	High school or technical secondary school = 1, College education = 0, Master's degree or above=0
	High school or technical secondary school = 0, College education = 1, Master's degree or above = 0
	High school or technical secondary school = 0, College education = 0, Master's degree or above = 1
Employment status	Employed full-time (Reference group)
	Employed part-time = 1, Other employment = 0
	Employed part-time = 0, Other employment = 1
Monthly family income (CNY)	≤ 5,000 (Reference group)
	5,001–10,000 =1, 10,001–15,000 =0, >15,000 = 0
	5,001–10,000 =0, 10,001–15,000 =1, >15,000 = 0
	5,001–10,000 =0, 10,001–15,000 =0, >15,000 = 1
Payment methods for healthcare costs	Self-funded (Reference group)
	Medical insurance = 1, Government-covered medical care = 0, Commercial insurance = 0, Medical insurance and commercial insurance = 0
	Medical insurance = 0, Government-covered medical care = 1, Commercial insurance = 0, Medical insurance and commercial insurance = 0
	Medical insurance = 0, Government-covered medical care=0, Commercial insurance = 1, Medical insurance and commercial insurance = 0
	Medical insurance = 0, Government-covered medical care = 0, Commercial insurance = 0, Medical insurance and commercial insurance = 1
Annual medical expenses of the child (CNY)	< 3,000 (Reference group)
	3,000–4,999 = 1, ≥ 5,000 = 0
	3,000–4,999 = 0, ≥ 5,000 = 1
PSSS total score	Continuous variables, direct inclusion
Family support	Continuous variables, direct inclusion
Friends support	Continuous variables, direct inclusion
Significant other support	Continuous variables, direct inclusion

**Table 7:** Multiple linear regression analysis of caregiver burden among parents of children with asthma comorbidities (*N* = 325).

**Variable**	**Category**	**Unstandardized regression coefficient**		**Standardized regression coefficient**	***t***	***P***	**Statistics of collinearity**		**95.0% CI**
		***B***	**SE**	β			**Tolerance of tolerance**	**VIF**	
(Constant)		75.121	3.295	–	22.798	0.000	–	–	68.663–81.579
Child's age (year)		−0.769	0.123	−0.279	−6.251	0.000	0.713	1.403	−1.010−0.528
Asthma course (year)	≥3	2.966	0.676	0.216	4.387	0.000	0.584	1.713	1.641–4.291
Asthma comorbidity course (year)	≥3	1.762	0.701	0.129	2.514	0.012	0.543	1.841	0.388–3.136
Number of asthma comorbidities	2 comorbidities	2.162	0.617	0.138	3.506	0.001	0.915	1.093	0.953–3.371
	≥3 comorbidities	10.272	2.447	0.165	4.199	0.000	0.917	1.091	5.476–15.068
Asthma control status	Poorly controlled	1.209	0.542	0.088	2.229	0.026	0.919	1.088	0.147–2.271
Employment status	Employed part-time	2.273	1.003	0.087	2.266	0.024	0.969	1.032	0.307–4.239
Payment methods for healthcare costs	Medical insurance and commercial insurance	−4.047	1.558	−0.102	−2.597	0.01	0.921	1.086	−7.100−0.994
Perceived social support scale		−0.721	0.068	−0.441	−10.528	0.000	0.811	1.233	−0.854−0.588

## 4 Discussion

### 4.1 Levels of caregiver burden among parents of children with asthma comorbidities

This study found that the total caregiver burden score for parents of children with asthma comorbidities was 40.44 ± 6.86, indicating a moderate level of burden. This level of burden was higher than that previously reported for parents of children with asthma alone (34), caregivers of children with general chronic diseases (35), and caregivers of children with diabetes (36). Several factors may explain these findings: compared to children with asthma only or general chronic diseases, the condition of children with asthma comorbidities is more complex. In addition to managing asthma symptoms, parents must also provide care for other comorbidities, significantly increasing the volume and difficulty of caregiving tasks. This often leads to a burden due to mental and physical exhaustion. The recurrent nature of asthma comorbidities may cause parents to feel uncertain about treatment effectiveness. Coupled with frequent medical visits and the financial pressure from overlapping treatment costs for multiple diseases, it can easily trigger emotions such as anxiety and fatigue (37), thereby increasing psychological burden.

Furthermore, this study revealed that 61.2% of parents of children with asthma comorbidities experienced a moderate level of caregiver burden. Among the dimensions of caregiver burden, personal burden ranked higher than role strain. This suggests that during the caregiving process, parents' physical and mental exhaustion is more pronounced than the burden derived from specific care tasks. The reasons for this may be that the complex condition of children with asthma comorbidities requires substantial time and energy investment in care. Parents often sacrifice personal rest, social activities, and even work, leading to long-term neglect of their own needs and accumulation of personal burden (38). At the same time, facing the potential recurrence of the child's illness or interactions among multiple diseases, parents are prone to self-blame, which further exacerbates psychological pressure at the personal level. Therefore, relevant departments can reduce the overall caregiver burden on parents by establishing temporary care support channels to provide short-term respite opportunities, implementing targeted psychological interventions to help alleviate negative emotions, and offering care pathway guidelines for comorbidities to simplify daily care processes.

### 4.2 Factors associated with caregiver burden among parents of children with asthma comorbidities

#### 4.2.1 Perceived social support

This study demonstrated that perceived social support among parents of children with asthma comorbidities is negatively correlated with caregiver burden, meaning that higher levels of social support are associated with lower levels of burden, which is consistent with previous studies (34, 39). Ding et al. (40) also noted that social support is a crucial factor influencing caregivers' care activities and supportive behaviors. High levels of social support can not only directly alleviate parents' physical, psychological, and financial pressures but also promote positive coping mechanisms, thereby enhancing mental health and reducing the risk of anxiety and depression (41). Additionally, access to resources such as medical information and care benefits can directly reduce care-related concerns and lessen physical and financial strain (42). Conversely, parents with low perceived social support are more likely to face caregiving alone due to a lack of external support, experiencing difficulties in obtaining emotional guidance and practical assistance, leading to an accumulated burden. This survey revealed that only 12.7% of parents of affected children reported a high level of perceived social support. Therefore, clinical institutions and communities should prioritize supporting populations with low social support by implementing targeted interventions. These may include conducting one-on-one assessments to identify specific deficiencies (e.g., lack of emotional or informational support), encouraging parents to proactively strengthen communication with relatives and friends and participate in mutual support groups for caregivers of children with chronic illnesses, and promoting the establishment of diversified community support platforms, such as organizing family support lectures and creating online experience-sharing groups. These efforts would help parents build a robust social support network, thereby enhancing their perceived social support and alleviating caregiver burden.

#### 4.2.2 Child's age

This study demonstrated that the age of the child was significantly associated with caregiver burden among parents. A negative correlation was observed between the child's age and the level of caregiver burden experienced by parents of children with asthma comorbidities, a result consistent with the report by Yang et al. (43). Older children demonstrated greater physiological and psychological maturity, enhanced self-care capacity, improved cognitive and self-management abilities, as well as decreased anxiety and resistance toward treatment and nursing care. These developments reduced the demand for parental assistance in daily care and disease management, thereby alleviating physical and psychological stress (43). In contrast, younger children were more prone to poor cooperation, distress behaviors such as crying, and limited self-management awareness due to their illness and therapeutic regimens, thereby increasing caregiving difficulty (44). Furthermore, parents of very young children assumed nearly complete responsibility for both daily living and medical care (45), which consumed considerable time and energy. This often resulted in diminished social participation, restricted access to informational and emotional support, and heightened perceptions of social isolation, collectively contributing to elevated caregiver burden (46). Therefore, it is suggested that relevant departments and healthcare institutions prioritize parents of young children with asthma comorbidities, implementing targeted interventions focusing on three aspects: competence, resources, and psychology. Collaborate with healthcare professionals to conduct practical training sessions, teaching parents how to use toys to guide their children's cooperation during treatment and symptom monitoring, while also distributing care manuals. For older children, provide simultaneous self-management education through interactive lessons to teach basic care skills, gradually reducing parental caregiving pressure. Utilize community-based platforms to establish short-term care support, organize mutual aid groups, and integrate lectures by psychologists to help parents share experiences and manage care-related stress.

#### 4.2.3 Payment methods for healthcare costs

This study demonstrated that the payment methods for healthcare costs were associated with lower levels of caregiver burden among parents of children with asthma comorbidities. Parents from families utilizing a combination of “medical insurance and commercial insurance” reported significantly lower levels of caregiver burden, a finding consistent with the results reported by Tu et al. (47). Several factors may explain these findings: the long-term treatment required for pediatric asthma comorbidities entails substantial medical expenditures. The dual coverage provided by medical and commercial insurance can complementarily offset these costs, significantly reducing out-of-pocket expenses for families. This reduction in financial pressure alleviates cost-related anxiety, thereby mitigating caregiver burden. In contrast, families relying solely on out-of-pocket payments or single medical insurance schemes face heightened economic and psychological strain. Out-of-pocket payers bear the full cost of treatment, while those with only basic medical insurance may find that coverage limitations fail to meet the comprehensive demands of multimorbidity management, as supported by previous studies (48, 49). Jiang et al. (48) suggested that the payment methods for healthcare costs influence caregivers' positive perceptions. Out-of-pocket payment imposes a heavy economic burden on caregivers, leading to a decline in their positive attitudes toward themselves and society. In contrast, insurance reimbursement can alleviate some of the financial pressure and have a protective effect on caregivers' physical and mental health. Therefore, it is suggested that multi-faceted intervention strategies be implemented at the national level: improve the healthcare security system, encourage families to adopt a combination of “medical insurance and commercial insurance,” and promote the establishment of specialized medical insurance programs for children with asthma (50). It is also essential to optimize reimbursement policies for the diagnosis and treatment of comorbidities to further reduce medical expenses. By alleviating the financial pressure on families, the caregiver burden on parents can be reduced.

#### 4.2.4 Number of asthma comorbidities

This study demonstrated that the number of comorbidities was significantly associated with caregiver burden among parents of children with asthma comorbidities, indicating that a higher number of comorbidities are associated with a greater level of caregiver burden. This suggests that the coexistence of multiple comorbidities may significantly increase caregiver burden by exacerbating the difficulty of asthma control and complicating treatment management processes. The underlying reason for this is that an increase in the number of comorbidities complicates the clinical condition of children with asthma (51). On one hand, parents must simultaneously address the dual care demands of asthma and multiple other diseases, resulting in an increased caregiving workload. On the other hand, potential interactions between different diseases may prolong treatment cycles and increase the frequency of medical visits, thereby amplifying parents' practical and psychological burdens. Additionally, investigations have demonstrated that the frequency of asthma attacks and emergency department visits significantly rises with an increasing number of comorbidities (52), and multiple comorbidities represent a major risk factor for poor asthma control (53). Other studies have also pointed out that asthma comorbidities directly contribute to a higher frequency of asthma deterioration (13). These negative changes in disease status ultimately translate into parental concerns regarding disease prognosis, further exacerbating the caregiver burden. Therefore, healthcare providers and community workers should prioritize parents of children with a higher number of comorbidities and regularly assess changes in their caregiver burden. Personalized care guidance plans can be developed for such families. For example, by simplifying multi-disease care processes and providing coordinated one-stop follow-up services to reduce the complexity of care. Additionally, collaboration with health insurance and social support systems should be strengthened to offer medical expense subsidies or assistance channels, thereby mitigating the compounded impact of financial pressure and alleviating parental caregiver burden.

#### 4.2.5 The disease course of asthma and asthma comorbidities

This study demonstrated that the disease course of asthma and asthma comorbidities was positively correlated with caregiver burden among parents, indicating that a longer disease course was associated with a higher level of burden, which is consistent with previous studies (54). Several factors may explain these findings: a longer disease course implies a prolonged period during which caregivers must invest time in caregiving (55). Over time, the continuous focus of time and energy on the child leads caregivers to gradually abandon their original lifestyles, as leisure and rest time are occupied, resulting in a cumulative increase in psychological load (56). As chronic conditions, asthma and its comorbidities require long-term management, sometimes throughout the child's life, and are prone to recurrence. During extended care, caregivers inevitably experience emotional and psychological burdens, often accompanied by anxiety regarding treatment efficacy (57). Additionally, a longer disease course often indicates a more complex clinical condition and greater difficulty in disease control, necessitating more frequent treatments. This not only increases medical expenses but may also lead to treatment resistance in children due to unmet long-term expectations. Consequently, parents must expend additional effort to guide and persuade their children to cooperate with treatment, further exacerbating the caregiver burden. Therefore, clinical institutions and community departments should prioritize families with long-term disease courses and implement stratified interventions. This can be achieved by establishing a case manager system for such families, where dedicated personnel track disease progression and streamline multidisciplinary diagnosis and treatment processes. Additionally, regular mutual support activities should be organized, involving psychological experts to provide emotional counseling, thereby helping parents alleviate long-term care pressure and ultimately reducing the caregiver burden on families across different stages of the disease.

#### 4.2.6 Asthma control status

This study demonstrated that the asthma control status of the children was significantly associated with caregiver burden, indicating that poorer asthma control was associated with greater caregiver burden among parents, which is consistent with previous findings (58). Furthermore, a correlation was observed between the degree of disease control in children and parental caregiving burden, with suboptimal disease management increasing the perceived burden among parents (59). Several factors may explain these findings: asthma comorbidities are a risk factor for decreased asthma control in children (60) and are closely associated with increased asthma exacerbations and poor control (61). This implies that parents must simultaneously manage both asthma and comorbid conditions, leading to increased care complexity and perceived burden. Furthermore, children with poorly controlled asthma often experience recurrent symptoms, reduced drug efficacy, and repeated hospitalizations, necessitating that parents invest more time and effort in closely monitoring symptoms and managing acute episodes. This prolonged state of high vigilance gradually accumulates psychological strain. Additionally, as the primary caregivers, parents also face dual pressures from both disease management and medical expenses (62).

Therefore, clinical institutions and community departments should prioritize families of children with poorly controlled asthma and implement targeted interventions. These may include collaborating with healthcare professionals to provide disease management training for parents, enhancing their ability to monitor symptoms and respond to emergencies; establishing a follow-up mechanism for asthma control to facilitate timely adjustment of treatment plans and reduce disease recurrence; and organizing regular psychological support and family mutual-aid activities to help alleviate parental anxiety, thereby mitigating the caregiver burden associated with poor asthma control.

#### 4.2.7 Employment status

This study demonstrated that the employment status of the parents was associated with their caregiver burden. Specifically, parents engaged in part-time employment reported relatively higher levels of caregiving burden. The study also indicated that poorer family economic status was associated with greater caregiver burden (63). The need for long-term treatment and regular follow-up visits for asthma comorbidities often leads to work absenteeism, reduced working hours, and income loss among caregivers, significantly increasing the financial strain on families. Under such economic pressure, some parents may resort to working overtime or taking on part-time jobs to supplement household income (64).

Several factors may explain these findings: asthma comorbidities require regular management, while part-time work often involves fragmented schedules, making it difficult to establish a stable care routine. Parents may need to frequently interrupt work to care for their children, leading to reduced work efficiency and compressed rest time. In cases of fluctuations in the child's condition, the time constraints of part-time work may force parents to face the dilemma of either losing income due to missed work or delaying necessary care. Moreover, the income stability and level from part-time work are generally lower than those of full-time employment, often insufficient to fully cover the long-term medical expenses and care-related costs of the child. This amplifies parents' concerns about inadequate care resources and intensifies the psychological burden. Additionally, these parents must balance part-time work, childcare, and daily household responsibilities such as supporting older adult(s) family members. Under multiple role pressures, they are prone to neglect their own health (65), remaining in a prolonged state of physical and mental exhaustion, which further exacerbates the caregiver burden. Therefore, it is suggested that policy and service-level interventions be implemented to provide more care-friendly employment options for parents, including remote work opportunities and flexible scheduling (66), to help them better balance occupational demands with caregiving responsibilities and ultimately reduce caregiver burden.

## 5 Limitations

This study has several limitations. First and most importantly, the cross-sectional nature of the study design precludes the determination of causal relationships between the identified factors and caregiver burden. The associations observed only indicate relationships at a single point in time. Second, the data were collected exclusively through self-reported questionnaires, which introduces the potential for self-report bias. For instance, parents' assessments of their psychological state, social support, and burden level may be influenced by social desirability or transient mood states. Third, the generalizability of the findings is limited by the convenience sampling method and the recruitment of participants from only two tertiary hospitals in a single province of China. The experiences of caregivers in other regions or healthcare settings may differ. Finally, while the study examined several key factors, it may not have encompassed all relevant variables, such as specific caregiver coping strategies or detailed clinical markers of disease severity.

Future research should therefore prioritize longitudinal or prospective cohort designs to establish temporal precedence and better infer causality. Expanding to multi-center, multi-regional studies with larger and more diverse samples is essential to enhance the external validity of the findings. Incorporating objective measures, such as healthcare utilization records or clinical biomarkers of asthma control, would help mitigate self-report bias. Furthermore, investigating a broader range of psychosocial variables, including resilience, caregiver self-efficacy, and family functioning, could provide a more comprehensive understanding of the determinants of caregiver burden.

## 6 Conclusion

In conclusion, this study demonstrates that parents of children with asthma comorbidities experience a considerable level of caregiver burden, with over 60% experiencing moderate burden. Factors associated with higher caregiver burden in this group include asthma course (≥3 years), number of asthma comorbidities (2 or ≥3 comorbidities), asthma comorbidity course (≥3 years), employment status (part-time employment), and poor asthma control. Conversely, perceived social support, the child's age, and having both medical and commercial insurance are associated with lower caregiver burden.

The caregiver burden status of these parents requires focused attention from clinical institutions, communities, and relevant authorities. Going forward, an intervention system can be built around the influencing factors: enhancing parents' perceived social support by establishing community mutual-aid platforms and organizing family support activities; developing integrated disease management plans for families of children with long disease duration, multiple comorbidities, or poorly controlled asthma, simplifying care procedures, and strengthening skill training; addressing role conflict issues among part-time parents by collaborating with communities and enterprises to provide care support and psychological counseling; and working with medical insurance departments to optimize medical security policies for children with asthma and comorbidities, expanding the coverage of “dual insurance.” Through multi-level collaboration involving families, medical institutions, communities, and policy sectors, alleviating the care burden of these parents will not only improve the caregivers' quality of life but also provide family-level safeguards for the children's standardized treatment and health outcomes.

## Data Availability

The original contributions presented in the study are included in the article/supplementary material, further inquiries can be directed to the corresponding authors.
